# Caesarean section trends in Catalonia between 2013 and 2017 based on the Robson classification system: A cross-sectional study

**DOI:** 10.1371/journal.pone.0234727

**Published:** 2020-06-16

**Authors:** Garazi Carrillo-Aguirre, Albert Dalmau-Bueno, Carlos Campillo-Artero, Anna García-Altés

**Affiliations:** 1 Master’s Degree Public Health, Pompeu Fabra University (UPF), Barcelona, Spain; 2 Department of Health, Health Evaluation and Quality Agency of Catalonia (AQuAS), Government of Catalonia, Barcelona, Catalonia, Spain; 3 Center for Research in Health and Economics, Pompeu Fabra University, IB-Salut, Barcelona, Spain; 4 Biomedical Research Networking Center for Epidemiology and Public Health (CIBERESP), Madrid, Spain; 5 Sant Pau Biomedical Research Institute (IIB Sant Pau), Barcelona, Spain; African Population and Health Research Center, KENYA

## Abstract

**Introduction:**

In Catalonia caesarean rates have always been analysed as a single percentage. The objective is to estimate caesarean section rates using the Robson classification in publicly funded hospitals in Catalonia between 2013 and 2017, considering sociodemographic, institutional and obstetric characteristics.

**Materials and methods:**

Cross-sectional population-based study in Catalonia including all women delivering within publicly funded hospitals between 2013–2017 (n = 210 020). The modified Robson classification distribution was estimated, the caesarean rate and the overall contribution, analysed for each year, and by confounders, through logistic regression models.

**Results:**

CS rates decreased steadily between 2013 and 2017 in Catalonia within publicly funded hospitals from 24.3% to 22.8% (cOR 0.92, 95% CI; 0.89 to 0.95). Once adjusted for changes in sociodemographic, institutional and obstetric characteristics the observed decline was even more pronounced (aOR 0.87, 95% CI; 0.84 to 0.90). Within the different groups of Robson once adjusted for confounders, groups 1+2 (aOR 0.88, 95% CI; 0.83 to 0.93), 3+4 (aOR 0.83, 95% CI; 0.78 to 0.89) and 10 (aOR 0.78, 95% CI; 0.68 to 0.90) presented a reduction in caesarean section rates, whereas group 5 showed no significant decrease (aOR 0.95, 95% CI; 0.87 to 1.03%).

**Conclusions:**

The decrease in caesarean section rates in Catalonia is more pronounced when adjusted for known confounders, suggesting retrospective overutilization of caesarean section and percentages of (in)adequacy in the past. In any case, it remains above the recommended by experts. Further efforts should be made to achieve optimum rates, including improvement on obstetric data collection

## Introduction

A Caesarean Section (CS) is a surgical procedure that, when performed for medical reasons, could save the life of a woman and her baby. However, it carries risks for both of them and therefore should only be considered when necessary [1,2].

The increase on the global rates of CS remains a continuing public health concern [3,4]. The World Health Organization (WHO) stated that priority should remain the provision of CS to all women in need, rather than the achievement of an ideal level [5]. In places where CS is universally available though, optimal rates should be expected (i.e. Catalonia). Not excepted of controversy, recent studies suggested rates between 10–20% of all births [6–9].

There are complex reasons behind the significant increase in CS [10], believed, in principle, to correlate with higher risk profiles of pregnant women and their babies [11]. However, Betrán et al. suggested that it responds to a multifactorial phenomenon including healthcare organisations as well as financial aspects among others [12].

During the years, the comparison of national rates has mask inequalities on access and practice. The WHO (2015) proposed the Robson classification as a global standard system for assessing, monitoring and comparing CS rates [5]. This classifies women into ten different groups, all mutually exclusive, and totally inclusive, based on obstetric characteristics [13].

In Catalonia, the CS rate have concerned health authorities for very long time. Monitoring of CS rate started in 1990 and different published National Health Plans have included the objective of CS reduction [14–18]. However, despite having also implemented several protocols and guidelines in regards to care during delivery and the publication of official reports comparing CS rates between hospitals [19–24], their impact is unknown due to a lack of exhaustive evaluation assessments. Rates have ranged between 22% and 32%, with considerable differences between the public and the private sectors, i.e. 22.3% to 35.9% for the year 2017 [25]. Maternal and neonatal mortality ratio has remained very low (MMR: 3.1, 2010–2014 [26] and NMR: 1.67, 2014–2017 [27]) and public hospitals do not provide CS under maternal request [28]. In this context, this analysis provides an exciting opportunity for a deeper understanding of the CS rate fluctuation.

Motivated by the opportunity that the Robson classification provides, this current study analyses CS trends between 2013 and 2017, in order to firstly, identify the groups of women with the highest contribution to CS rates, and secondly, observe changes in the total CS rate by adjusting for sociodemographic, institutional and obstetric characteristics. The results hope to provide valuable information for the establishment of public health benchmarks for maternal and neonatal health programs, as well as the design of policies and guidelines.

## Material and methods

### Study design and participants

**Fig 1:**
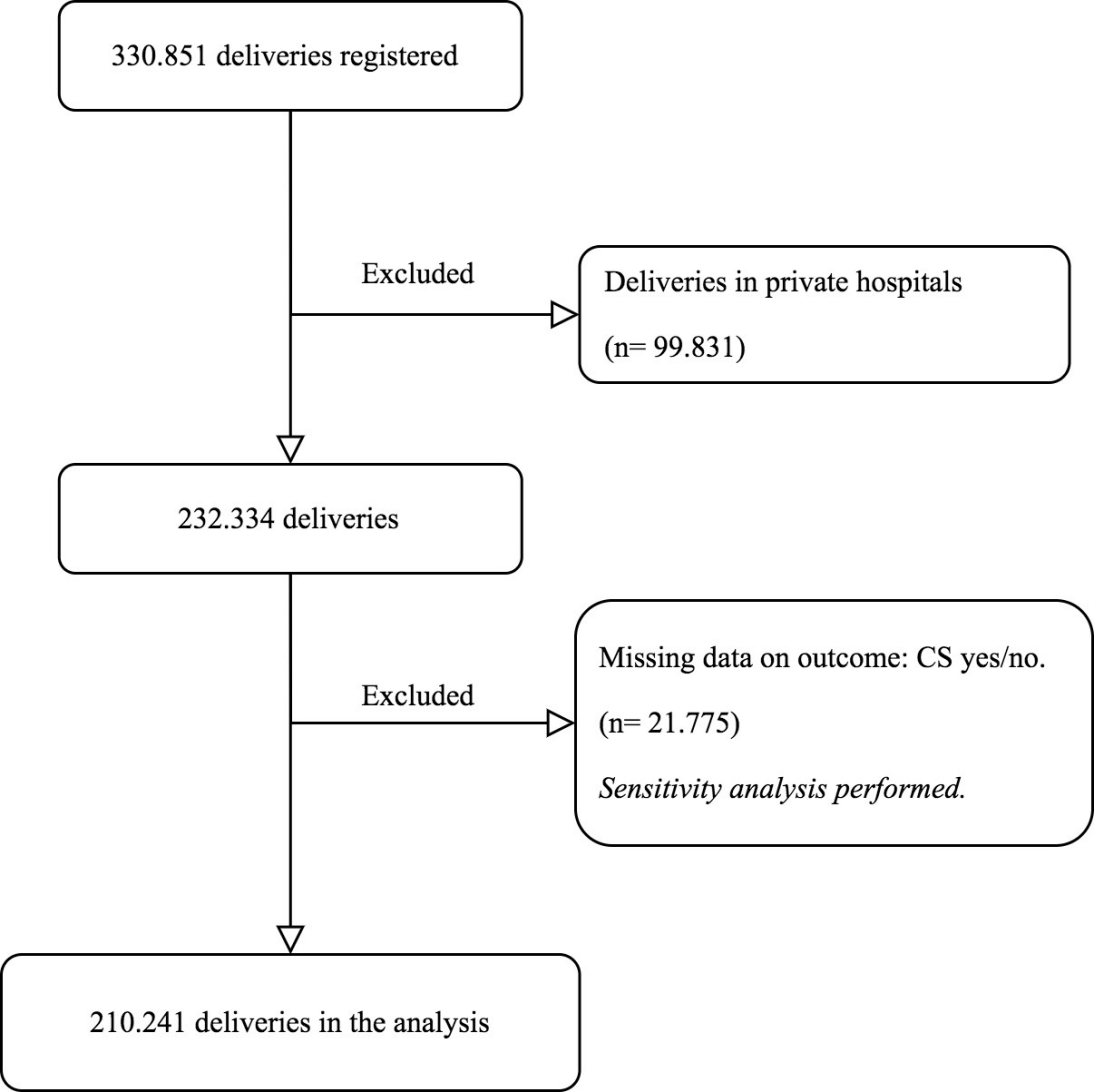
Flowchart of the population included in the study.

A retrospective cross-sectional trend study was conducted in the Spanish region of Catalonia. The population included women delivering between 1^st^ January 2013 and 31^st^ December 2017. From the total 330,851 (100%) deliveries occurred after 22 weeks gestation, only those that occurred at the 44 publicly funded hospitals offering maternity care in Catalonia were included. As a result, 231,020 (69.83%) deliveries met the eligibility criteria for this research. However, information regarding type of delivery, whether it was vaginal or caesarean section, was considered a *sine qua non* variable for the study. Thus, missing data in 9.1% of the women regarding outcome (vaginal/CS), reduced the potential sample size to 210,241 (Fig 1).

### Details of ethics approval

A governmental structure called PADRIS exists within the Catalan healthcare administration. PADRIS is an analytical data programme for research and innovation in health. The purpose of the structure is to make data available to scientific communities to promote research, innovation, and evaluation in health, with the aim of reusing and exchanging the data generated by the health system in accordance with the legal and regulatory framework. The programme PADRIS ensures that all data made available to researchers is fully anonymised. As workers within the Catalan healthcare administration, we are provided data by PADRIS, and therefore are never given access to identifiable information. Thus, the study did not involve any data collection, requiring neither human participants nor patient consent. For that reason, and due to the use of existing anonymised data for research, the study was exempt from institutional review committee approval. It is the standard way of proceeding in the healthcare administration to systematically check the quality of the healthcare providers in our context.

### Data sources

Three data sources were employed: (1) Minimum Basic Data Set (*Conjunt Mínim Básic de Dades*, CMBD); (2) the Central Registry of Insured Persons (*Registro Central de personas Aseguradas*, RCA); and (3) the Clinic Station for Primary Care (*Estació Clínica d’ Atenció Primària*, E-CAP).

Individuals on the registries were given a personal anonymised code that allowed linkage between databases. The different databases were then merged using this anonymised code.

1. CMBD is an administrative registry subject to Ministerial regulation containing exhaustive data regarding hospital discharges. All hospitals are required to provide information regarding hospital activity and diagnosis (ICD-9-CM at the time of the current study) [29]. This registry allowed access to information regarding type of delivery and the majority of variables required for the Robson classification.

2. RCA collects personal data of all those insured by the CatSalut (Catalan National Health System), including income level and employment status [30], enabling access to socioeconomic status.

3. E-CAP is a computerized system used within the primary healthcare facilities. This enabled access to details of Body Mass Index (BMI) and parity [31].

### Definition of variables

The primary outcome for this study was delivery by CS. The following variables were required to create the variable Robson classification: parity, gestational age, foetal presentation, previous CS, number of foetuses, and onset of labour [32]. The variable foetal presentation was not recorded within the databases, so it had to be created ad hoc, thus considering cephalic presentation in the absence of breech or transverse lie. A modified classification was constructed due to onset of labour not being collected in any of the databases.

An adapted Robson classification was created formed of eight mutually exclusive categories. This combined groups 1 and 2, and 3 and 4, with the rest remaining unchanged: nulliparous, singleton cephalic pregnancy, ≥ 37 weeks’ gestation (groups 1+2); multiparous without a previous uterine scar, with singleton, cephalic pregnancy, ≥ 37 weeks’ gestation (groups 3+4); previous CS, singleton, cephalic, ≥ 37 weeks’ gestation (Group 5); all nulliparous with a single breech (Group 6); all multiparous with a single breech, including previous CS (Group 7); all multiple pregnancies, including previous CS (Group 8); all women with a single pregnancy in transverse or oblique lie (including those with previous CS (Group 9); all singleton, cephalic, < 37 weeks’ gestation pregnancies, including previous CS (Group 10).

The following variables were also included: age at the time of delivery, nationality, socioeconomic status, hospital level, BMI and maternal complications. For the variable nationality The World Bank classification was selected [33]. The variable socioeconomic status is a set variable within the database that arises through pharmaceutical co-pay estimations. There are four co-payment groups: 1) those exempt from co-payment, (disadvantaged population; individuals receiving some form of universal pension scheme, those who no longer receive unemployment allowance, or those who no longer receive unemployment benefit and do not qualify for unemployment allowance); 2) those with annual earnings of less than 18,000€; 3) those with annual earnings between 18,000€ and 100,000€; and 4) those with annual earnings over 100,000€. This is predetermined by the system and does not allow further disaggregation. The hospital level follows an order of complexity, based on the care that the pregnant woman and her baby might require. Thus, even though all hospitals will be caring for women with low risk pregnancies, the more complex the pregnancy becomes the higher the level of the hospital in which she will be cared for. Level IA being the less medicalised hospitals, and IIIB the most, offering medical attention from other specialties if required [34]. The variable maternal complication was created ad hoc and included any of the following diagnoses that had to be recorded prior initiating the labour process within this pregnancy: uterine rupture, placenta praevia, pre-eclampsia, gestational diabetes, diabetes mellitus, heart disease, hypertension, hepatic disease, viral disease, anaemia, renal disease and epilepsy. A code recorded at CMBD that includes different diagnosis called “disease that complicates the pregnancy” was also included (For further information S1 Table).

### Data analysis

#### Missing data

Missing data relating to the different variables ranged between 0.2 and 32%, with CS showing an overall level of 6%. Maternal age accounted for 0.2%; nationality 1.2%; socioeconomic status 0.2%; healthcare facility 1.6%; and BMI 32%. To minimize loss of statistical power, BMI was not included in the final model and a separate sensibility analysis was undertaken. An analysis of the missing data concerning type of delivery was also undertaken to determine any bias related to the response rates.

#### Statistical analysis

Characteristics of the women included in the study were reported for each year, along with the proportion of women delivered by CS. The following were analysed for each specific year and modified Robson classification: the relative size of the obstetric population (% = n of women in the group/total N women delivered x 100), total CS rate (% = n of CS in the group/total N of women in the group x 100) and the absolute contribution to the total CS rate (% = n of CS in the group/total N of women delivered). The crude change between 2013 and 2017 was calculated for each of the above.

Two logistic regression models were used for the analysis between the principal variable (CS) and the remainder of the variables. Crude and adjusted odds ratios were estimated with their correspondent 95% confidence intervals (CI) and p-values. Following the same analytical strategy, maintaining the CS as dependent variable, through ten regression logistic models, crude and adjusted odds ratios were also estimated for each year and for each Robson group for each year.

Finally, the performance of the logistic models applied to estimate time trends were assessed. On the one hand, the area under the receiver operating curve (ROC) was estimated to determine the discriminatory capacity and on the other hand, the calibration belt p-value for internal validation. This analysis pretended to establish a reliable baseline. The aim of the study was to analyse CS trends and the intention was not to build a predictive model for CS, although the possibility was explored. Stata software version 14 was used to perform the analysis.

## Results

A significant decrease was identified in CS rates between 2013 and 2017, from 24.30% to 22.80% (see Table 1). The majority of the women on the population sample were Spanish (62.43%), with an income of less than 18.000€ (76.15%). Their average age was 30 (SD 5.6), with most having singleton, cephalic births at term. Some characteristics changed slightly over the years, with the proportion of births to women aged thirty-five or over increasing by 5.23% and an overall significant increase of women with a BMI above 30. In addition, the decrease in the proportion of nulliparous women and increase in multiparous women with previous CS were statistically significant. There was also an increase in the number of women presenting a diagnosis potentially complicating the pregnancy.

**Table 1:** Socio-demographic and obstetric characteristics in the study population for each year, 2013–2017.

	Year 2013			Year 2014			Year 2015			Year 2016			Year 2017		
	n = 43145			n = 42397			n = 41825			n = 41275			n = 41599		
	n	%	95% CI	n	%	95% CI	n	%	95% CI	n	%	95% CI	n	%	95% CI
**Type of delivery**															
Vaginal	32659	75.7	75.29–76.10	32134	75.79	75.38–76.20	31802	76.04	75.62–76.44	31526	76.37	75.96–76.78	32114	77.2	76.79–77.60
CS	10486	24.3	23.90–24.71	10263	24.21	23.80–24.62	10023	23.96	23.56–24.38	9752	23.63	23.22–24.04	9485	22.8	22.40–23.21
**Parity**															
Nuliparous	16486	38.21	37.75–38.67	15647	36.91	36.45–37.37	14790	35.36	34.90–35.82	14097	34.15	33.69–34.61	14274	34.31	33.86–34.77
Multiparous	26659	61.79	61.33–62.25	26750	63.09	62.63–63.55	27035	64.64	64.18–65.10	27181	65.85	65.39–66.31	27325	65.69	65.23–66.14
**Previous CS**															
No	39054	90.52	90.24–90.79	38266	90.26	89.97–90.54	37496	89.65	89.35–89.94	36889	89.37	89.07–89.66	37116	89.22	88.92–89.52
Yes	4091	9.48	9.21–9.76	4131	9.74	9.46–10.03	4329	10.35	10.06–10.65	4389	10.63	10.34–10.93	4483	10.78	10.48–11.08
**Maternal age years**															
<20	1319	3.06	2.90–3.22	1218	2.87	2.72–3.04	1192	2.85	2.69–3.01	1159	2.81	2.65–2.97	1159	2.79	2.63–2.95
20–24.9	5040	11.68	11.38–11.99	4673	11.02	10.73–11.32	4498	10.75	10.46–11.06	4473	10.84	10.54–11.14	4393	10.56	10.27–10.86
25–29.9	10590	24.55	24.14–24.95	10370	24.46	24.05–24.87	9940	23.77	23.36–24.18	9600	23.26	22.85–23.67	9470	22.76	22.36–23.17
30–34.9	15028	34.83	34.38–35.28	14618	34.48	34.03–34.93	14008	33.49	33.04–33.95	13695	33.18	32.73–33.64	13632	32.77	32.32–33.22
35–39.9	9205	21.34	20.95–21.72	9503	22.41	22.02–22.81	9960	23.81	23.41–24.22	9895	23.97	23.56–24.39	10269	24.69	24.27–25.10
≥40	1963	4.55	4.36–4.75	2015	4.75	4.55–4.96	2227	5.32	5.11–5.54	2453	5.94	5.72–6.18	2676	6.43	6.20–6.67
**Nationality**															
Spain	27116	62.85	62.39–63.30	26428	62.33	61.87–62.80	26314	62.91	62.45–63.38	25503	61.78	61.31–62.25	25886	62.23	61.76–62.69
Rest of Europe and Central Asia	2591	6.01	5.78–6.23	2643	6.23	6.01–6.47	2712	6.48	6.25–6.72	2707	6.56	6.32–6.80	2835	6.82	6.57–7.06
Middle East and North Africa	5830	13.51	13.19–13.84	5442	12.84	12.52–13.16	5430	12.98	12.66–13.31	5559	13.47	13.14–13.80	5634	13.54	13.22–13.88
Latin América and Caribbean	4368	10.12	9.84–10.41	4555	10.74	10.45–11.04	4246	10.15	9.86–10.45	4302	10.42	10.13–10.72	3889	9.35	9.07–9.63
East Asia and Pacific	992	2.3	2.16–2.45	1007	2.38	2.23–2.52	802	1.92	1.79–2.05	841	2.04	1.90–2.18	790	1.9	1.77–2.03
Subsaharian Africa	1138	2.64	2.49–2.79	1130	2.67	2.51–2.82	1163	2.78	2.63–2.94	1118	2.71	2.55–2.87	1075	2.58	2.43–2.74
South Asia	1094	2.54	2.39–2.69	1152	2.72	2.56–2.88	1133	2.71	2.56–2.87	1216	2.95	2.78–3.11	1463	3.52	3.34–3.70
North America	16	0.04	0.02–0.06	40	0.09	0.07–0.13	25	0.06	0.04–0.09	32	0.08	0.05–0.11	27	0.06	0.04–0.09
**Socioeconomic status**															
Disadvantaged population	2516	5.83	5.61–6.06	2123	5.01	4.80–5.22	2200	5.26	5.05–5.48	2349	5.69	5.47–5.92	2270	5.46	5.24–5.68
Income <18.000	32238	74.72	74.31–75.13	32517	76.7	76.29–77.10	32393	77.45	77.05–77.85	31703	76.8	76.39–77.21	31246	75.11	74.69–75.53
Income 18.000–100.000	8369	19.4	19.03–19.77	7740	18.26	17.89–18.63	7208	17.23	16.87–17.60	7202	17.45	17.08–17.82	8057	19.37	18.99–19.75
Income >100.000	22	0.05	0.03–0.08	17	0.04	0.02–0.06	24	0.06	0.04–0.09	24	0.06	0.04–0.09	26	0.06	0.04–0.09
**Healthcare facility**															
Level IA	13375	31	30.56–31.44	12757	30.09	29.65–30.53	12660	30.27	29.83–30.71	12140	29.41	28.97–29.85	11813	28.4	27.96–28.83
Level IIA	7070	16.39	16.04–16.74	7032	16.59	16.23–16.94	6694	16	15.65–16.36	6356	15.4	15.05–15.75	6406	15.4	15.05–15.75
Level IIB	6666	15.45	15.11–15.79	6602	15.57	15.23–15.92	6557	15.68	15.33–16.03	6591	15.97	15.62–16.32	7107	17.08	16.72–17.45
Level IIIA	10566	24.49	24.08–24.90	10526	24.83	24.42–25.24	10547	25.22	24.80–25.64	10577	25.62	25.20–26.05	10442	25.1	24.69–25.52
Level IIIB	5468	12.67	12.36–12.99	5480	12.93	12.61–13.25	5367	12.83	12.51–13.16	5614	13.6	13.27–13.93	5831	14.02	13.68–14.35
**Fetal presentation**															
Cephalic	41833	96.96	96.79–97.12	41123	97	96.83–97.16	40503	96.84	96.67–97.00	40040	97	96.83–97.16	40324	96.94	96.76–97.10
Breech	1122	2.6	2.45–2.76	1067	2.52	2.37–2.67	1154	2.76	2.60–2.92	1061	2.57	2.42–2.73	1090	2.62	2.47–2.78
Oblique/Transverse	190	0.44	0.38–0.51	207	0.49	0.42–0.56	168	0.4	0.34–0.47	177	0.43	0.37–0.50	185	0.44	0.38–0.51
**Gestational age**															
≥ 37 weeks term	40492	93.85	93.62–94.08	39822	93.93	93.69–94.15	39383	94.16	93.93–94.38	38811	94.02	93.79–94.25	39194	94.22	93.99–94.44
<37 weeks preterm	2653	6.15	5.92–6.38	2575	6.07	5.85–6.31	2442	5.84	5.62–6.07	2467	5.98	5.75–6.21	2405	5.78	5.56–6.01
**Pregnancy complication**															
No	28616	66.33	65.88–66.77	27274	64.33	63.87–64.79	27054	64.68	64.22–65.14	26254	63.6	63.14–64.07	26266	63.14	62.68–63.60
Yes	14529	33.67	33.23–34.12	15123	35.67	35.21–36.13	14771	35.32	34.86–35.78	15024	36.4	35.93–36.86	15333	36.86	36.40–37.32
**Number of neonates**															
Singleton	42360	98.18	98.05–98.30	41589	98.09	97.96–98.22	41047	98.14	98.01–98.27	40426	97.94	97.79–98.07	40781	98.03	97.90–98.16
Multiple	785	1.82	1.70–1.95	808	1.91	1.78–2.04	778	1.86	1.73–1.99	852	2.06	1.93–2.21	818	1.97	1.84–2.10
**Robson classification**															
Group 1+2	14505	33.62	33.17–34.07	13761	32.46	32.01–32.91	12940	30.94	30.50–31.38	12379	29.99	29.55–30.43	12542	30.15	29.71–30.59
Group 3+4	20335	47.13	46.66–47.60	20425	48.18	47.70–48.65	20616	49.29	48.81–49.77	20678	50.09	49.61–50.58	20723	49.82	49.33–50.30
Group 5	4210	9.76	9.48–10.04	4203	9.91	9.63–10.20	4304	10.29	10.00–10.59	4316	10.46	10.16–10.76	4455	10.71	10.41–11.01
Group 6	532	1.23	1.13–1.34	499	1.18	1.08–1.28	526	1.26	1.15–1.37	431	1.04	0.95–1.15	450	1.08	0.98–1.19
Group 7	487	1.13	1.03–1.23	469	1.11	1.01–1.21	525	1.26	1.15–1.37	507	1.23	1.12–1.34	524	1.26	1.15–1.37
Group 8	785	1.82	1.70–1.95	808	1.91	1.78–2.04	778	1.86	1.73–1.99	852	2.06	1.93–2.21	818	1.97	1.84–2.10
Group 9	159	0.37	0.31–0.43	178	0.42	0.36–0.49	145	0.35	0.29–0.41	140	0.34	0.29–0.40	163	0.39	0.33–0.46
Group 10	2132	4.94	4.74–5.15	2054	4.84	4.64–5.05	1991	4.76	4.56–4.97	1975	4.78	4.58–4.99	1924	4.63	4.43–4.83
	**n = 29804**			**n = 29602**			**n = 29257**			**n = 29272**			**n = 29338**		
**Body Mass Index BMI**															
<18.5	533	1.79	1.64–1.95	590	1.99	1.84–2.16	586	2	1.85–2.17	580	1.98	1.82–2.15	605	2.06	1.90–2.23
18.5–24.9	13496	45.28	44.72–45.85	13504	45.62	45.05–46.19	13292	45.43	44.86–46.00	13085	44.7	44.13–45.27	12732	43.4	42.83–43.97
25–29.9	10100	33.89	33.35–34.43	9894	33.42	32.89–33.96	9753	33.34	32.80–33.88	9669	33.03	32.49–33.57	9847	33.56	33.02–34.11
30–34.9	4047	13.58	13.19–13.97	4018	13.57	13.19–13.97	4034	13.79	13.39–14.19	4252	14.53	14.12–14.93	4364	14.87	14.47–15.29
35–39.9	1205	4.04	3.82–4.27	1178	3.98	3.76–4.21	1192	4.07	3.85–4.31	1240	4.24	4.01–4.47	1345	4.58	4.35–4.83
≥40	423	1.42	1.29–1.56	418	1.41	1.28–1.55	400	1.37	1.24–1.51	446	1.52	1.39–1.67	445	1.52	1.38–1.66

### Determinants of caesarean sections

During the study period, 50.009 women were delivered by CS. Rates increased in relation to maternal age, women under twenty with a rate of 14.59%, while those over forty 37.18% (S2 Table). Adjusting for sociodemographic, institutional and obstetric factors, the age differences remain similar. In particular, women between 35 and 40 presented a 37% higher chance of CS, and women over 40, twice the likelihood of having a CS than women aged between 25 and 30 (Table 2).

**Table 2:** Association between caesarean section and socio-demographic and obstetric characteristics.

	Crude odd ratio			Adjusted odd ratio		
	OR	95% CI	p-value	OR	95% CI	p-value
**Maternal age years**						
<20	0.65	0.60–0.70	<0.001	0.64	0.59–0.69	<0.001
20–24.9	0.79	0.76–0.82	<0.001	0.82	0.78–0.86	<0.001
25–29.9	1			1		
30–34.9	1.16	1.13–1.20	<0.001	1.1	1.07–1.14	<0.001
35–39.9	1.5	1.46–1.55	<0.001	1.37	1.33–1.42	<0.001
≥40	2.25	2.15–2.35	<0.001	2	1.90–2.10	<0.001
**Nationality**						
Spain	1			1		
Rest of Europe and Central Asia	0.84	0.80–0.88	<0.001	0.86	0.82–0.90	<0.001
Middle East and North Africa	0.71	0.69–0.73	<0.001	0.77	0.74–0.80	<0.001
Latin America and Caribbean	1.16	1.12–1.20	<0.001	1.16	1.12–1.21	<0.001
East Asia and Pacific	0.65	0.60–0.70	<0.001	0.7	0.64–0.76	<0.001
Sub-Saharian Africa	1.15	1.09–1.23	<0.001	1.21	1.12–1.29	<0.001
South Asia	1.19	1.13–1.27	<0.001	1.15	1.07–1.22	<0.001
North America	0.51	0.32–0.83	0.006	0.49	0.29–0.82	0.007
**Socioeconomic status**						
Disadvantaged population	0.84	0.81–0.89	<0.001	0.9	0.86–0.96	<0.001
Income <18.000 €	1			1		
Income 18.000–100.000 €	1.07	1.04–1.09	<0.001	0.87	0.85–0.90	<0.001
Income >100.000 €	0.73	0.46–1.18	0.002	0.47	0.27–0.80	0.006
**Healthcare facility**						
Level IA	1			1		
Level IIA	1.03	1.00–1.06	0.065	0.95	0.92–0.99	0.001
Level IIB	1.06	1.02–1.09	0.001	0.94	0.90–0.97	<0.001
Level IIIA	1.2	1.17–1.23	<0.001	1.09	1.05–1.12	<0.001
Level IIIB	1.37	1.33–1.42	<0.001	1.07	1.03–1.11	<0.001
**Pregnancy complication**						
No	1			1		
Yes	1.43	1.41–1.46	<0.001	1.44	1.41–1.47	<0.001
**Robson classification**						
Group 1+2	1			1		
Group 3+4	0.38	0.37–0.39	<0.001	0.36	0.35–0.37	<0.001
Group 5	3.87	3.75–4.00	<0.001	3.55	3.43–3.67	<0.001
Group 6	141.44	107.94–185.34	<0.001	145.07	110.67–190.16	<0.001
Group 7	78.92	64.44–96.66	<0.001	78.29	63.89–95.94	<0.001
Group 8	5.53	5.18–5.91	<0.001	4.91	4.59–5.26	<0.001
Group 9	86.62	59.38–126.36	<0.001	74.67	51.14–109.03	<0.001
Group 10	1.75	1.67–1.83	<0.001	1.59	1.52–1.66	<0.001

In comparison to Spanish women (24.45%), higher rates of CS were found in women from sub-Saharan Africa (27.19%), Latin America and the Caribbean (27.27%) and South Asia (27.89%) (S2 Table). These groups remained similar after adjusting for confounders (Table 2).

The variable of socioeconomic level revealed that women with an income of over 18.000€ showed less probability of CS. Differences were also noted relating to the complexity level of the hospital, with Level IIIB undertaking the highest number and Level IA the least (Level IIIB compared to Level IA, crude OR 1.37, 95% CI 1.33–1.42). However, these differences can be explained when adjusting for confounders (Level IIIB compared to Level IA OR 1.07, 95% CI 1.03–1.11). Moreover, as expected, women with any diagnosis potentially complicating the pregnancy had a higher probability of CS, even when adjusting for other confounders (OR 1.44, 95% CI 1.41–1.47) (Table 2).

### Trends over time in caesarean section rates among the modified Robson groups

**Fig 2:**
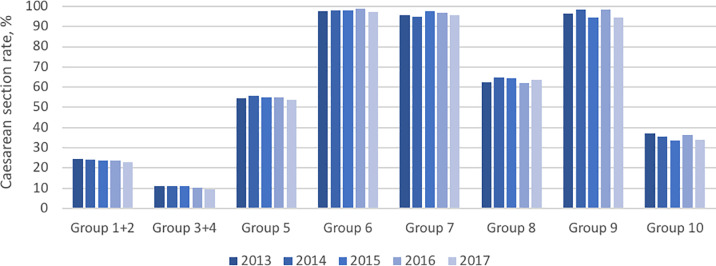
Caesarean section rate.

**Fig 3:**
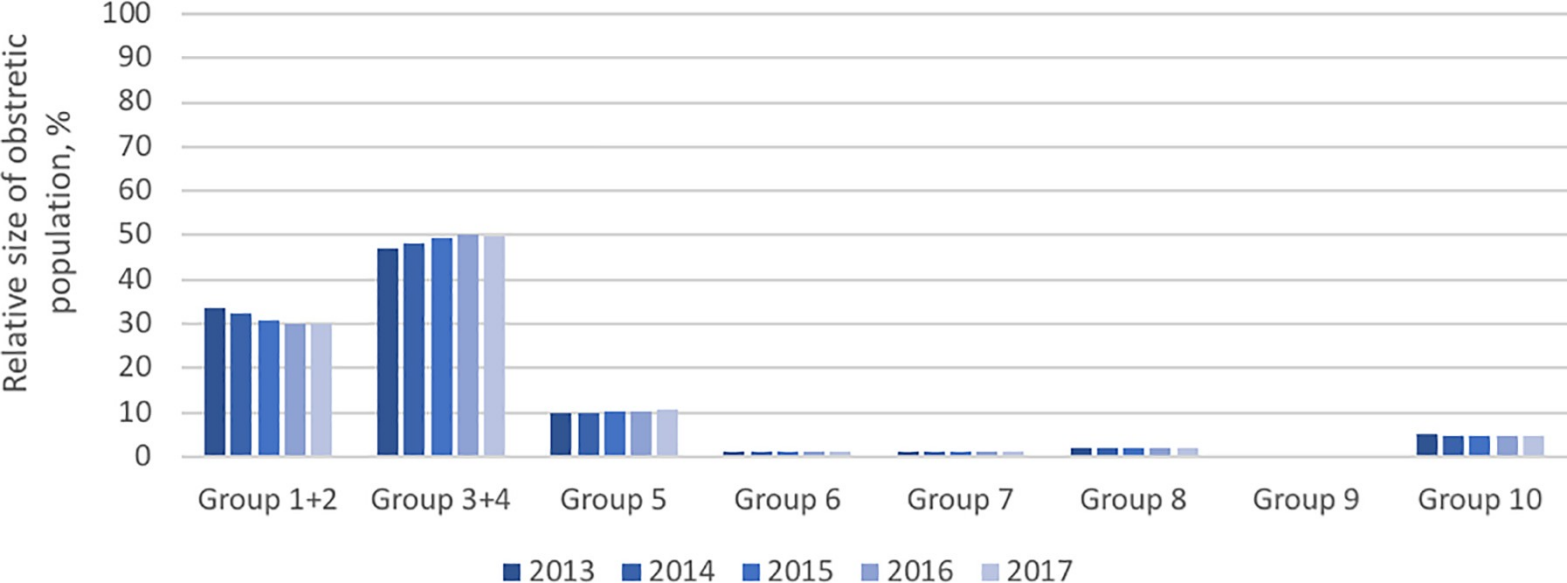
Relative size of the obstetric population.

**Fig 4:**
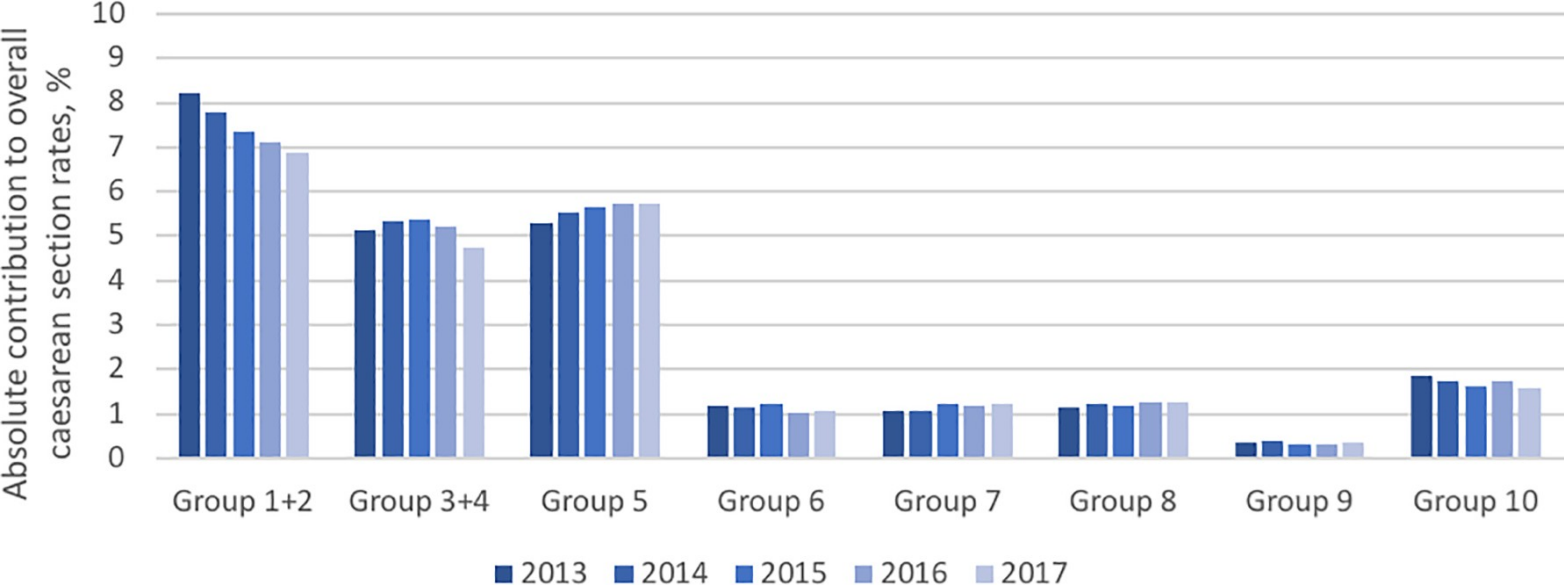
Absolute contribution to overall caesarean section rates.

The overall CS rate steadily declined between 2013 and 2017. The following figures show each of the obstetric groups according to the modified Robson classification during the five years of the study period: Fig 2, the CS rate; Fig 3, the relative size of the obstetric population; and Fig 4, the absolute contribution to the overall CS rate.

Within the population sample, the majority of women carried single babies at term in cephalic presentation and belonged to groups 1+2 and 3+4 (31.45% and 48.88%, respectively), followed by multiparous women with a uterine scar (10.22%) and women with premature babies (4.79%).

The CS rate was lowest for groups 1+2 and 3+4 and highest (i.e. almost 100%) for groups 6, 7 and 9. Group 5 had an average rate of 54.73%; Group 8 63.33% and Group 10 35.34% (see Fig 2). Most groups demonstrated a subtle downwards tendency in CS rates observed in the graph.

The major contributors to the absolute CS rates were groups 1+2, 3+4 and 5. The considerable reduction in group 1+2 was due to the reduced group size and CS rates. By contrast, group 5 showed an increased overall contribution due to the increasement on the size.

It has been established that high-risk pregnancies are a risk factor for CS. The initial analyses revealed that the probability of CS in 2017 was, in comparison to 2013, reduced by 8% (OR 0.92, 95% CI 0.89–0.95) (Table 3). However, the profile of women described in Table 1 revealed a significant increase in known risk factors for CS (i.e. age or pregnancy complications). Thus, the analysis adjusted by confounders showed that there was even less probability in comparison to 2013 i.e. 13% (OR 0.87, 95% CI 0.84–0.90). The significant reduction in CS rates was already observed in 2015 (OR 0.96, CI 95% 0.92–0.99) and maintained throughout 2016 (OR 0.93, 95% CI 0.90–0.97) (Table 3).

**Table 3:** Time trends in caesarean section rates in all women and by groups of Robson.

	Calibration belt p-value‾	Area under the ROC curve	2013⌂			2014				2015				2016				2017			
		Area CI95%	CS rate CI95%		OR	CS rate CI95%		OR CI95%		CS rate CI95%		OR CI95%		CS rate CI95%		OR CI95%		CS rate CI95%		OR CI95%	
Crude																					
All	0.915	0.75 0.74–0.75	24.3	23.90–24.71	1	24.21	23.80–24.61	0.99	0.96–1.03	23.96	23.56–24.37	0.98	0.95–1.01	23.63	23.22–24.04	0.96	0.93–0.99*	22.8	22.40–23.20	0.92	0.89–0.95***
Robson 1+2	1	0.51 0.50–0.51	24.5	23.80–25.20	1	23.98	23.27–24.69	0.97	0.92–1.03	23.77	23.04–24.50	0.96	0.91–1.02	23.7	22.95–24.44	0.96	0.90–1.01	22.86	22.12–23.59	0.91	0.86–0.97***
Robson 3+4	1	0.51 0.50–0.51	10.94	10.51–11.37	1	11.05	10.62–11.48	1.01	0.95–1.08	10.88	10.45–11.30	0.99	0.93–1.06	10.41	10.00–10.83	0.95	0.89–1.01	9.49	9.09–9.89	0.85	0.80–0.91**
Robson 5	1	0.51 0.50–0.51	54.35	52.84–55.85	1	55.86	54.36–57.37	1.06	0.98–1.16	55.04	53.56–56.53	1.03	0.94–1.12	54.89	53.40–56.37	1.02	0.94–1.11	53.58	52.12–55.04	0.97	0.89–1.06
Robson 10	1	0.50 0.50,-0.51	37.2	35.14–39.25	1	35.54	33.47–37.61	0.93	0.82–1.06	33.7	31.63–35.78	0.86	0.76–0.98*	36.2	34.08–38.32	0.96	0.84–1.09	33.89	31.77–36.00	0.87	0.76–0.98*
**Adjusted**																					
All†	0.01	0.77 0.76–0.77	24.56	24.20–24.92	1	24.29	23.93–24.65	0.98	0.95–1.02	23.9	23.54–24.26	0.96	0.92–0.99*	23.59	23.22–23.95	0.93	0.90–0.97***	22.56	22.20–22.92	0.87	0.84–0.90***
Robson 1+2•	0.17	0.59 0.59–0.60	24.92	24.23–25.62	1	24	23.29–24.70	0.95	0.90–1.00	23.72	22.99–24.44	0.93	0.88–0.99*	23.52	22.78–24.26	0.92	0.87–0.98**	22.61	21.89–23.33	0.88	0.83–0.93***
Robson 3+4•	0.86	0.59 0.58–0.59	11.14	10.70–11.57	1	11.05	10.62–11.48	0.99	0.93–1.05	10.81	10.39–11.23	0.97	0.91–1.03	10.33	9.92–10.75	0.92	0.86–0.98**	9.45	9.05–9.85	0.83	0.78–0.89***
Robson 5•	0.74	0.59 0.58–0.59	54.64	53.15–56.13	1	55.97	54.48–57.46	1.06	0.97–1.15	55.15	53.68–56.62	1.02	0.94–1.11	54.69	53.22–56.17	1	0.92–1.09	53.29	51.84–54.74	0.95	0.87–1.03
Robson 10•	0.76	0.58 0.57–0.58	37.97	36.01–39.92	1	36.12	34.15–38.09	0.91	0.80–1.05	34.11	32.13–36.10	0.83	0.73–0.95**	35.47	33.48–37.47	0.89	0.77–1.01	32.9	30.92–34.88	0.78	0.68–0.90***

⌂Reference group

† Adjusted for maternal age, nationality, socioeconomic status, healthcare facility, pregnancy complication, delivery year and modified Robson classification.

• Adjusted for maternal age, nationality, socioeconomic status, healthcare facility, pregnancy complication, delivery year and modified Robson classification.

‾ Goodness of fit assuming polynomial relationship

*p-value <0.05

**p-value <0.01

***p-value<0.001

Adjusted odds ratios for the different Robson groups were also estimated, apart from groups 6 to 9, which presented very small sample size. Women in groups 1+2 had a 12% (OR 0.88, 95% CI 0.83–0.93) lower chance of having a CS, with women in groups 3+4 experiencing 17% (OR 0.83, 95% CI 0.78–0.89). Women having premature babies (Group 10) presented a 22% lower chance, although wide CI were obtained due to the sample size (OR 0.78, 95% CI 0.68–0.90). Nevertheless, the reduction was not observed in every group, since Group 5 demonstrated no significant differences for any of the years (OR 0.95, 95% CI 0.87–1.03, 2017) (Table 3).

It was thus established that fewer CS were performed in Catalonia between 2013 and 2017. Within the years that showed the greatest decrease (p-value <0.05) 2015, 2016, 2017 in CS, up to 2.319 CS (95% CI, 1399–3239) were avoided.

Model validation showed that the crude analysis which included all women was well calibrated and had a high grade of discrimination (calibration belt p-value: 0.915, ROC 0.75, 95% 0.74–0.75), however once the model was adjusted the calibration was dramatically affected (p-value 0.01) while the discriminant ability remained similar (ROC 0.77, IC 95% 0.76–0.77). The analysis of the different Robson groups showed good calibration but little discriminatory effect.

## Discussion

### Main findings

This study identified a significant decrease in CS rates in publicly funded hospitals in Catalonia between 2013 and 2017. When adjusted the CS rates for well-known risk factors, the reduction was even more pronounced. Considering that the characteristics of women are evolving into a higher risk profiles, this means that in equal conditions there are now less CS performed.

It is important to note that nulliparous (Group 1+2), multiparous (Group 3+4) and multiparous women with previous CS (Group 5) contributed most to the overall CS rate, suggesting that possibly are the groups that present larger margins for interventions on the aim of reducing final percentages of CS.

### Strengths

The most significant contribution of this study is the analysis of the individual level data from administrative registries. It enabled the analysis of CS rates for each group of Robson, rather than for the population, as simplified to a single number. Also, the large sample provided an important statistical power to the evidence shown and is innovative since includes adjustment variables not yet examined together in this type of research before.

A considerable difference in CS rates was identified between public and private settings in Catalonia [25]. The focus on deliveries in publicly funded hospitals excluded CS on demand, thus avoiding any possible bias.

To the best of the current researcher’s knowledge, this is the first time the Robson classification has been implemented at a population level, within the Mediterranean countries, employing real world data and with consideration of so many confounders. The results could potentially be generalized to other Southern European countries with a similar healthcare framework.

### Limitations

The categorisation of the 10 groups of Robson was constrained by not having the variable onset of labour recorded, therefore a modified version of the Robson classification had to be created. Also, the registry does not collect some other variables which they had to be created ad hoc, i.e. foetal presentation, which was considered cephalic in the absence of a diagnosis of breech or transverse lie. When performing the crosscheck analysis with the measures for data collection quality suggested at the Robson Implementation Manual [32], the parameters in general showed consistency with those proposed. However, having to create the variable presentation ad hoc implied that not all of them were actually transverse, because CS for Group 9 did not end up being 100%. Also, creating variables ad hoc implied a lack of unclassified cases, which is considered another parameter to measure data collection quality.

The variable BMI had many omissions and therefore could not be included as an adjusting factor. However, the sensibility analysis showed a similar decrease in CS rates (S3 Table). The analysis of the missing data on the primary outcome revealed more nulliparous than multiparous women (S4 Table). However, in view of the small percentage (6%) it was believed that it did not imply deliberate avoidance by hospitals of reporting CS. Furthermore, it was considered that due to the large study sample, results would not have changed significantly. As well as the variable of “pregnancy complication” that since relies on the provider’s clinical diagnosis, could be prone to bias.

This study sample tended to under-represent the highest socioeconomic category, due to private care, but in general, found no differences in CS rates between extreme socioeconomic levels. The numbers suggested that more Catalan nulliparous women attended the private sector, as this sample was constituted of 35.81% nulliparous and 64.19% multiparous, while national statistics showed about 50% [27].

Regarding the statistical analysis, the purpose of the study hampered the possibility of applying a multi-level approach. Although it is realistic to think that same women could have delivered more than once within the study period, the magnitude of the population sample should still provide relevant and valuable results.

### Interpretation

Following adjustment for individual risk factors, CS rates decreased in Catalonia between 2013 and 2017. However, comparison with other countries and aiming for optimal rates suggests scope for improvement [26,35].

Our study founds similar patterns to that observed in other European countries, groups 1 to 5 are the once contributing the most to the final CS rate [35]. However, attention should be paid to the reduced proportion of nulliparous women in the study sample, since this made the highest contribution to the overall CS rate and would had been higher in the absence of private care.

In addition, the evidence suggests that optimising CS rates requires focusing attention on groups 1 to 5. However, the ability to prioritize interventions as a result of our study is compromised by the inability to determine the onset of labour.

CS rates in Group 5 (i.e. women with previous CS) reflected the obstetric practice of previous years. It is particularly linked to CS performed on nulliparous women, and will therefore, if this continues to decrease, be reflected in Group 5. Also suggests that trial of labour following CS (TOLAC) should be prioritized in favour of optimum levels [36].

Rates for women with breech presentations remained the same in our study (groups 6 and 7). Following the Term Breech Trials results, CS for breech rapidly increased worldwide [37]. Spanish guidelines have recently upheld considering vaginal birth acceptable under some circumstances [38,39], with some hospitals in Catalonia now resuming them, although it will take time for numbers to reflect any change. Facilitating the external cephalic version, is in any case the preferred option, also for transverse presentations (group 9) [40–42].

The reason behind the overall decrease remains unclear. The protocol to promote natural birth [43], the adequacy of indications, the creation of an adjusted index for CS [44] or growing evidence of the risks that implies the CS [45] could only be some of the reasons behind. In addition, the fourth-wave of feminism, international organisations on birth-rights together with local organisations or growing eco on the media regarding obstetric violence, could have also influenced this reduction [46,47].

Furthermore, the application of systematic Robson classification has been suggested to contribute on the decrease of CS rates. In line with this, interventions and indications should also be audited. CS rates can be safely reduced by applying multifaceted strategies combining audit and feedback, implementing guidelines on mandatory second opinion or educating physicians by local opinion leaders [1,48]. In addition, midwife-led continuity models of care have also proved beneficial, including reducing CS for low risk women [49–51]. In Catalonia, one out of the seven projected midwifery-led units is already functioning. However, midwife ratios in Spain continue to be one of the lowest across European countries [52].

This analysis hopes to provide useful information to the public, public health experts and health professionals, however, does not intend to replace in any case the original Robson classification. It should only be a temporary tool until all needed variables are systematically recorded.

## Conclusion

This study found a significant decrease in CS rates in Catalonia between 2013–2017. Provides evidence that group 1+2, 3+4 and 10 are the groups that have shown the highest reduction after adjusting for confounders and suggests retrospective overutilization of CS and percentages of (in)adequacy in the past. Special attention should be paid to groups 1 to 5 since they imply the biggest contributors to the overall CS rates, and any reduction would imply a considerable reduction in the total rate.

The reasons behind remains unclear and further efforts should be made as rates remain above optimum levels. Including the Robson classification as a systematic way to analyse CS could be very useful to compare, assess and analyse data and prioritize, however all variables including onset of labour are necessary. Hence, policy makers should give urgent attention to the collection of perinatal data.

## Supporting information

S1 Checklist STROBE checklist. (DOCX)

S1 Table Diagnostic codes included in the variable pregnancy complication. (DOCX)

S2 Table Bivariate analysis of socio-demographic and obstetric characteristics and type of delivery. (DOCX)

S3 Table Time trends in caesarean section rates including the variable BMI by groups of Robson. (DOCX)

S4 Table Socio-demographic and obstetric characteristics of study population by type of delivery. (DOCX)

## References

[1] Chen I, Opiyo N, Tavender E, Mortazhejri S, Rader T, Petkovic J, et al Non-clinical interventions for reducing unnecessary caesarean section. Cochrane Database Syst Rev [Internet]. 2018 9 28 [cited 2019 May 2];9:CD005528 Available from: http://www.ncbi.nlm.nih.gov/pubmed/30264405 10.1002/14651858.CD005528.pub3 30264405 PMC6513634

[2] Villar J, Carroli G, Zavaleta N, Donner A, Wojdyla D, Faundes A, et al Maternal and neonatal individual risks and benefits associated with caesarean delivery: multicentre prospective study. BMJ [Internet]. 2007 11 17 [cited 2019 Jan 18];335(7628):1025 Available from: http://www.ncbi.nlm.nih.gov/pubmed/17977819 10.1136/bmj.39363.706956.55 17977819 PMC2078636

[3] Boerma T, Ronsmans C, Melesse DY, Barros AJD, Barros FC, Juan L, et al Global epidemiology of use of and disparities in caesarean sections. Lancet [Internet]. 2018 10 [cited 2018 Nov 17];392(10155):1341–8. Available from: https://linkinghub.elsevier.com/retrieve/pii/S0140673618319287 10.1016/S0140-6736(18)31928-7 30322584

[4] Betrán AP, Ye J, Moller A-B, Zhang J, Gülmezoglu AM, Torloni MR. The Increasing Trend in Caesarean Section Rates: Global, Regional and National Estimates: 1990–2014. Zeeb H, editor. PLoS One [Internet]. 2016 2 5 [cited 2019 Apr 22];11(2):e0148343 Available from: http://www.ncbi.nlm.nih.gov/pubmed/26849801 10.1371/journal.pone.0148343 26849801 PMC4743929

[5] Betran AP, Torloni MR, Zhang JJ, Gülmezoglu AM. WHO statement on caesarean section rates [Internet]. Vol. 123, BJOG: An International Journal of Obstetrics and Gynaecology. 2016 [cited 2019 Apr 22]. p. 667–70. Available from: https://apps.who.int/iris/bitstream/handle/10665/161442/WHO_RHR_15.02_eng.pdf;jsessionid=05AB78E8403CF090AFCFD9FB79994350?sequence=1 26681211 10.1111/1471-0528.13526 PMC5034743

[6] Molina G, Weiser TG, Lipsitz SR, Esquivel MM, Uribe-Leitz T, Azad T, et al Relationship Between Cesarean Delivery Rate and Maternal and Neonatal Mortality. JAMA [Internet]. 2015 12 1 [cited 2019 Jan 14];314(21):2263 Available from: http://jama.jamanetwork.com/article.aspx?doi=10.1001/jama.2015.15553 26624825 10.1001/jama.2015.15553

[7] Betran AP, Torloni MR, Zhang J, Ye J, Mikolajczyk R, Deneux-Tharaux C, et al What is the optimal rate of caesarean section at population level? A systematic review of ecologic studies. Reprod Health [Internet]. 2015 12 21 [cited 2018 Nov 17];12(1):57 Available from: http://reproductive-health-journal.biomedcentral.com/articles/10.1186/s12978-015-0043-6 26093498 10.1186/s12978-015-0043-6 PMC4496821

[8] Ye J, Zhang J, Mikolajczyk R, Torloni M, Gülmezoglu A, Betran A. Association between rates of caesarean section and maternal and neonatal mortality in the 21st century: a worldwide population-based ecological study with longitudinal data. BJOG An Int J Obstet Gynaecol [Internet]. 2016 4 [cited 2018 Nov 17];123(5):745–53. Available from: http://doi.wiley.com/10.1111/1471-0528.13592 10.1111/1471-0528.13592 PMC5014131 26331389

[9] Ye J, Betrán AP, Guerrero Vela M, Souza JP, Zhang J. Searching for the Optimal Rate of Medically Necessary Cesarean Delivery. Birth [Internet]. 2014 9 [cited 2019 Jan 14];41(3):237–44. Available from: http://www.ncbi.nlm.nih.gov/pubmed/24720614 10.1111/birt.12104 24720614

[10] Hyde MJ, Modi N. The long-term effects of birth by caesarean section: The case for a randomised controlled trial. Early Hum Dev [Internet]. 2012 12 [cited 2018 Nov 20];88(12):943–9. Available from: http://linkinghub.elsevier.com/retrieve/pii/S0378378212002198 10.1016/j.earlhumdev.2012.09.006 23036493

[11] Barber EL, Lundsberg LS, Belanger K, Pettker CM, Funai EF, Illuzzi JL. Contributing Indications to the Rising Cesarean Delivery Rate. Obstet Gynecol [Internet]. 2011 7 [cited 2019 Jan 18];118(1):29–38. Available from: http://www.ncbi.nlm.nih.gov/pubmed/21646928 10.1097/AOG.0b013e31821e5f65 21646928 PMC3751192

[12] Betrán AP, Temmerman M, Kingdon C, Mohiddin A, Opiyo N, Torloni MR, et al Interventions to reduce unnecessary caesarean sections in healthy women and babies. Lancet [Internet]. 2018 10 [cited 2019 Jan 16];392(10155):1358–68. Available from: https://linkinghub.elsevier.com/retrieve/pii/S0140673618319275 10.1016/S0140-6736(18)31927-5 30322586

[13] Souza J, Gülmezoglu A, Lumbiganon P, Laopaiboon M, Carroli G, Fawole B, et al Caesarean section without medical indications is associated with an increased risk of adverse short-term maternal outcomes: the 2004–2008 WHO Global Survey on Maternal and Perinatal Health. BMC Med [Internet]. 2010 12 10 [cited 2018 Nov 17];8(1):71 Available from: http://bmcmedicine.biomedcentral.com/articles/10.1186/1741-7015-8-71 21067593 10.1186/1741-7015-8-71 PMC2993644

[14] Social D de S i S. Pla de salut de Catalunya 1996–1998 [Internet]. 1997. Available from: http://salutweb.gencat.cat/web/.content/_departament/pla-de-salut/Plans-de-salut-anteriors/Pla-de-salut-de-Catalunya-1996-1998/documents/problemes_salutmaternitat98.pdf

[15] Generalitat de Catalunya. Departament de Salut. Pla de salut de Catalunya 2002–2005. Departament de Salut [Internet]. [cited 2019 Jun 19]. Available from: http://salutweb.gencat.cat/ca/el_departament/Pla_salut/plans-de-salut-anteriors/pla-de-salut-de-catalunya-2002-2005/

[16] Servei Català de la Salut. Pla de salut de la Regió Sanitària Barcelona a l ‘ horitzó 2010 Volum II les polítiques de salut [Internet]. 2010 [cited 2019 Jun 19]. Available from: https://catsalut.gencat.cat/web/.content/minisite/catsalut/catsalut_territori/barcelona/coneix_la_regio_sanitaria_barcelona/pla_territorial_de_salut/pla_de_salut_rsb_vol02.pdf

[17] Sanitari De Barcelona C. Pla de salut territorial [Internet]. 2012 [cited 2019 Jun 19]. Available from: http://www.csb.cat/wp-content/uploads/2015/04/Pla-de-salut-CSB-2011-2015.pdf

[18] Catalunya G de; D de S. Pla de Salut 2016–2020 [Internet]. Pla de Salut de Catalunya 2016–2020. 2016. 161 p. Available from: http://salutweb.gencat.cat/web/.content/home/el_departament/Pla_salut/pla_salut_2016_2020/Documents/Pla_salut_Catalunya_2016_2020.pdf

[19] Generalitat de Catalunya. Departamento de Salud. Protocolo para la asistencia natural al parto normal Biblioteca de Cataluña. Datos CIP Protocolo para la asistencia natural al parto normal Bibliografía [Internet]. 2007 [cited 2019 Jun 18]. Available from: https://www.elpartoesnuestro.es/sites/default/files/recursos/documents/protocolo_asistencia_parto_normal_cataluna_2007.pdf

[20] Cossio MLT, Giesen LF, Araya G, Pérez-Cotapos MLS, Vergara RL, Manca M, et al Guía para embarazadas. General Catalunya Dep Salut [Internet]. 2009 [cited 2019 Jan 20];XXXIII(2):81–7. Available from: http://www.anaelbusto.info/guiaembarcataluna.pdf

[21] Pública DG de S. Protocol de seguiment de l’embaràs a Catalunya [Internet]. Generalitat de Catalunya. Departament de Salut. Barcelona; 2018 [cited 2019 Jan 20]. Available from: http://salutpublica.gencat.cat/web/.content/minisite/aspcat/promocio_salut/embaras_part_puerperi/protocol_seguiment_embaras/protocol-seguiment-embaras-2018.pdf

[22] Agència de Qualitat i Avaluació Sanitàries de Catalunya. Central de Resultats [Internet]. Barcelona; 2015. Available from: http://observatorisalut.gencat.cat/web/.content/minisite/observatorisalut/ossc_central_resultats/informes/fitxers_estatics/CdR_Hospitals_dades_2015.pdf

[23] Spanish Ministry of Health. Strategy for Assitance at Normal Childbirth in the National Health System 2007. 2008; Available from: http://www.msssi.gob.es/organizacion/sns/planCalidadSNS/pdf/equidad/estrategiaPartoNormalEnglish.pdf

[24] Ministerio de Sanidad y Política Social. Gobierno de España. Guía de práctica clínica de atención al parto normal [Internet]. 2010. 103 p. Available from: http://www.guiasalud.es/GPC/GPC_472_Parto_Normal_Osteba_compl.pdf%0Ahttp://www.msssi.gob.es/organizacion/sns/planCalidadSNS/pdf/InformeFinalEAPN_revision8marzo2015.pdf

[25] Jané Checa M, Vidal Benedé MJ, Tomás Bonodo Z, Maresma Soler M. Indicadors de salut perinatal a Catalunya. Any 2017 [Internet]. Barcelona; 2018 [cited 2018 Nov 22]. Available from: http://canalsalut.gencat.cat/ca/professionals/vigilancia-epidemiologica/vigilancia-perinatal/

[26] EURO-PERISTAT. European Perinatal Health Report: http://www.europeristat.com. 2015; Available from: www.europeristat.com

[27] Generalitat de Catalunya. Idescat. Anuario estadístico de Cataluña. Indicadores de natalidad [Internet]. 2019 [cited 2019 Apr 28]. Available from: https://www.idescat.cat/pub/?id=aec&n=287&lang=es

[28] Sociedad Española de Ginecologia y Obstetricia (SEGO). documentoconsensoSEGO.pdf—Google Drive [Internet]. [cited 2019 Nov 5]. Available from: https://drive.google.com/file/d/0ByuDDZFNh88AZGNjMGY5NDctMDYzNi00OGM5LWJkZjMtZmM3MDc2ZmM2ZWMx/view

[29] Ministerio de Sanidad C y BS. Conjunto Mínimo Básico de Datos—Hospitalización. 2019 [cited 2019 May 3]; Available from: http://www.iasist.com/es/2181/Conjunto-Minimo-Basico-de-Datos-CMBD

[30] Generalitat de Catalunya. Registre central de població del CatSalut. CatSalut. Servei Català de la Salut [Internet]. 2019 [cited 2019 May 3]. Available from: https://catsalut.gencat.cat/ca/proveidors-professionals/registres-catalegs/registres/central-poblacio/

[31] Generalitat de Catalunya. eCAP. Departament de Salut [Internet]. 2018 [cited 2019 May 3]. Available from: http://salutweb.gencat.cat/ca/ambits_actuacio/linies_dactuacio/tecnologies_informacio_i_comunicacio/ecap/

[32] World Health Organization (WHO). Robson criteria implementation manual. 2017.

[33] The World Bank. World Bank Country and Lending Groups–World Bank Data Help Desk. World Bank [Internet]. 2017 [cited 2019 Apr 14];1–8. Available from: https://datahelpdesk.worldbank.org/knowledgebase/articles/906519-world-bank-country-and-lending-groups

[34] Direcció General de Planificació i Avaluació. Pla Estratègic d’Ordenació de l’Atenció Maternoinfantil als Hospitals de la Xarxa Hospitalària d’Utilització Pública a Catalunya. Barcelona; 2008.

[35] Pyykönen A, Gissler M, Løkkegaard E, Bergholt T, Rasmussen SC, Smárason A, et al Cesarean section trends in the Nordic Countries—a comparative analysis with the Robson classification. Acta Obstet Gynecol Scand [Internet]. 2017 5 [cited 2019 Apr 28];96(5):607–16. Available from: http://doi.wiley.com/10.1111/aogs.13108 28176334 10.1111/aogs.13108

[36] SEGO. Parto vaginal tras cesárea Protocolos Asistenciales en Obstetricia [Internet]. 2010 [cited 2019 May 2]. Available from: https://www.elpartoesnuestro.es/sites/default/files/recursos/documents/sego_protocolo_pvdc_2010.pdf

[37] Hannah ME, Hannah WJ, Hewson SA, Hodnett ED, Saigal S, Willan AR. Planned caesarean section versus planned vaginal birth for breech presentation at term: a randomised multicentre trial. Term Breech Trial Collaborative Group. Lancet (London, England) [Internet]. 2000 10 21 [cited 2019 May 2];356(9239):1375–83. Available from: http://www.ncbi.nlm.nih.gov/pubmed/11052579 10.1016/s0140-6736(00)02840-3 11052579

[38] Sociedad Española de Obstetricia y Ginecología. External cephalic version. Progresos Obstet y Ginecol [Internet]. 2015 8 1 [cited 2019 May 2];58(7):337–40. Available from: http://linkinghub.elsevier.com/retrieve/pii/S0304501314002775

[39] Azria E, Le Meaux J-P, Khoshnood B, Alexander S, Subtil D, Goffinet F, et al Factors associated with adverse perinatal outcomes for term breech fetuses with planned vaginal delivery. Am J Obstet Gynecol [Internet]. 2012 10 [cited 2019 May 2];207(4):285.e1–285.e9. Available from: http://www.ncbi.nlm.nih.gov/pubmed/23021690 23021690 10.1016/j.ajog.2012.08.027

[40] Salzer L, Nagar R, Melamed N, Wiznitzer A, Peled Y, Yogev Y. Predictors of successful external cephalic version and assessment of success for vaginal delivery. J Matern Neonatal Med [Internet]. 2015 1 2 [cited 2019 May 2];28(1):49–54. Available from: http://www.ncbi.nlm.nih.gov/pubmed/24593778 10.3109/14767058.2014.900749 24593778

[41] Royal College of Obstetricians and Gynaecologists. External Cephalic Version and Reducing the Incidence of Term Breech Presentation. BJOG An Int J Obstet Gynaecol [Internet]. 2017 [cited 2019 May 2];124(7):e178–92. Available from: https://www.rcog.org.uk/en/guidelines-research-services/guidelines/gtg20a/10.1111/1471-0528.14466 28299867

[42] The American College of Obstetrician and Gynaecologists. Practice Bulletin No. 161. Obstet Gynecol [Internet]. 2016 2 [cited 2019 May 2];127(2):e54–61. Available from: http://insights.ovid.com/crossref?an=00006250-201602000-00051 10.1097/AOG.0000000000001312 26942387

[43] Generalitat de Catalunya. Departamento de Salud. Protocolo para la asistencia natural al parto normal Biblioteca de Cataluña. Datos CIP Protocolo para la asistencia natural al parto normal Bibliografía. Dirección General de Salud Pública, editor. Barcelona; 2007.

[44] Agència de Qualitat i Avaluació Sanitàries de Catalunya. Àmbit Hospitalari. Dades del 2015. 2016; Available from: https://scientiasalut.gencat.cat/bitstream/handle/11351/3165/central_resultats_ambit_hospitalari_dades_2015.pdf?sequence=1&isAllowed=y

[45] Sandall J, Tribe RM, Avery L, Mola G, Visser GH, Homer CS, et al Short-term and long-term effects of caesarean section on the health of women and children. Lancet [Internet]. 2018 10 13 [cited 2019 Jun 20];392(10155):1349–57. Available from: http://www.ncbi.nlm.nih.gov/pubmed/30322585 10.1016/S0140-6736(18)31930-5 30322585

[46] Birth rights: protecting human rights in childbirth. 2010 [cited 2019 Jun 20];(November):2010. Available from: http://www.birthrights.org.uk/

[47] DONA LLUM–Associació Catalana per un Part Respectat–Treballem per a aconseguir uns parts més respectats i segurs, i uns naixements més feliços. [Internet]. [cited 2019 Jun 20]. Available from: https://www.donallum.org/

[48] Chaillet N, Dumont A. Evidence-Based Strategies for Reducing Cesarean Section Rates: A Meta-Analysis. Birth [Internet]. 2007 3 [cited 2019 May 2];34(1):53–64. Available from: http://www.ncbi.nlm.nih.gov/pubmed/17324180 10.1111/j.1523-536X.2006.00146.x 17324180

[49] McLachlan H, Forster D, Davey M-A, Farrell T, Flood M, Shafiei T, et al The effect of primary midwife-led care on women’s experience of childbirth: results from the COSMOS randomised controlled trial. BJOG An Int J Obstet Gynaecol [Internet]. 2016 2 [cited 2019 May 2];123(3):465–74. Available from: http://doi.wiley.com/10.1111/1471-0528.13713 10.1111/1471-0528.13713 26498455

[50] McLachlan H, Forster D, Davey M, Farrell T, Gold L, Biro M, et al Effects of continuity of care by a primary midwife (caseload midwifery) on caesarean section rates in women of low obstetric risk: the COSMOS randomised controlled trial. BJOG An Int J Obstet Gynaecol [Internet]. 2012 11 [cited 2019 May 2];119(12):1483–92. Available from: http://www.ncbi.nlm.nih.gov/pubmed/22830446 10.1111/j.1471-0528.2012.03446.x 22830446

[51] Sandall J, Soltani S, Gates S, Shennan A, Devane D. Midwifeled continuity models versus other models of care for childbearing women (Review). Cochrane database Syst Rev. 2016;4(10):46–67. 10.1002/14651858.CD004667.pub5 PMC8663203 27121907

[52] Eurostat. File:Practising midwives, 2011 and 2016 (per 100 000 inhabitants) HLTH18.png—Statistics Explained [Internet]. 2018 [cited 2019 May 2]. Available from: https://ec.europa.eu/eurostat/statistics-explained/index.php?title=File:Practising_midwives,_2011_and_2016_(per_100_000_inhabitants)_HLTH18.png

