# Supplementary material for: Caesarean section trends in Catalonia between 2013 and 2017 based on the Robson classification system: A cross-sectional study
Source: PLoS One. 2020 Jun 16;15(6):e0234727. doi: 10.1371/journal.pone.0234727 (PMC7297373; doi:10.1371/journal.pone.0234727)
Supplement: S1 Table — (DOCX) [file pone.0234727.s002.docx]

**Table S1** Diagnostic codes included in the variable pregnancy complication.

| **Uterine rupture** | |
| --- | --- |
| ICD9dx66501 | Rupture of uterus before onset of labour, delivered, with or without mention of antepartum condition |
| ICD9dx66503 | Rupture of uterus before onset of labour, antepartum condition or complication |
| ICD9dx66511 | Rupture of uterus during labour, delivered, with or without mention of antepartum condition |
| **Placenta praevia** | |
| ICD9dx64101 | Placenta previa without haemorrhage, delivered, with or without mention of antepartum condition |
| ICD9dx64111 | Haemorrhage from placenta previa, delivered, with or without mention of antepartum condition |
| ICD9dx46121 | Premature separation of placenta, delivered, with or without mention of antepartum condition |
| ICD9dx64131 | Antepartum haemorrhage associated with coagulation defects, delivered, with or without mention of antepartum condition |
| ICD9dx64181 | Other antepartum haemorrhage, delivered, with or without mention of antepartum condition |
| ICD9dx64191 | Unspecified antepartum haemorrhage, delivered, with or without mention of antepartum condition |
| **Preeclampsia** | |
| ICD9dx64201 | Benign essential hypertension complicating pregnancy, childbirth, and the puerperium, delivered, with or without mention of antepartum condition |
| ICD9dx64211 | Hypertension secondary to renal disease, complicating pregnancy, childbirth, and the puerperium, delivered, with or without mention of antepartum condition |
| ICD9dx64221 | Other pre-existing hypertension, complicating pregnancy, childbirth, and the puerperium, delivered, with or without mention of antepartum condition |
| ICD9dx64231 | Transient hypertension of pregnancy, delivered, with or without mention of antepartum condition |
| ICD9dx64241 | Mild or unspecified pre-eclampsia, delivered, with or without mention of antepartum condition |
| ICD9dx64251 | Severe pre-eclampsia |
| ICD9dx64261 | Eclampsia, delivered, with or without mention of antepartum condition |
| ICD9dx64271 | Pre-eclampsia or eclampsia superimposed on pre-existing hypertension, delivered, with or without mention of antepartum condition |
| ICD9dx64290 | Unspecified hypertension complicating pregnancy, childbirth, or the puerperium, unspecified as to episode of care or not applicable |
| ICD9dx64291 | Unspecified hypertension complicating pregnancy, childbirth, or the puerperium, delivered, with or without mention of antepartum condition |
| **Gestational diabetes** | |
| ICD9dx64881 | Abnormal glucose tolerance of mother, delivered, with or without mention of antepartum condition |
| **Diabetes mellitus** | |
| ICD9dx64801 | Diabetes mellitus of mother, complicating pregnancy, childbirth, or the puerperium, delivered, with or without mention of antepartum condition |

| **Cardiovascular disease** | |
| --- | --- |
| ICD9dx64861 | Other cardiovascular diseases of mother, delivered, with or without mention of antepartum condition |
| ICD9dx64851 | Congenital cardiovascular disorders of mother, delivered, with or without mention of antepartum condition |
| **Liver disease** | |
| ICD9dx2870 | Allergic purpura |
| ICD9dx2871 | Qualitative platelet defects |
| ICD9dx2872 | Other nonthrombocytopenic purpuras |
| ICD9dx2873 | Primary thrombocytopenia, unspecified |
| ICD9dx2875 | Thrombocytopenia, unspecified |
| ICD9dx2878 | Other specified haemorrhagic conditions |
| ICD9dx2879 | Unspecified haemorrhagic conditions |
| ICD9dx28730 | Primary thrombocytopenia, unspecified |
| ICD9dx28731 | Immune thrombocytopenic purpura |
| ICD9dx28732 | Evans' syndrome |
| ICD9dx28733 | Congenital and hereditary thrombocytopenic purpura |
| ICD9dx28739 | Other primary thrombocytopenia |
| ICD9dx28741 | Posttransfusion purpura |
| ICD9dx28749 | Other secondary thrombocytopenia |
| **Viral disease** | |
| ICD9dx042 | Human immunodeficiency virus [HIV] disease |
| ICD9dx64761 | Other viral diseases in the mother, delivered, with or without mention of antepartum condition |
| **Anaemia** | |
| ICD9dx64821 | Anemia of mother, delivered, with or without mention of antepartum condition |
| **Renal disease** | |
| ICD9dx40490 | Hypertensive heart and chronic kidney disease, unspecified, without heart failure and with chronic kidney disease stage I through stage IV, or unspecified |
| ICD9dx64620 | Unspecified renal disease in pregnancy, without mention of hypertension, unspecified as to episode of care or not applicable |
| ICD9dx64621 | Unspecified renal disease in pregnancy, without mention of hypertension, delivered, with or without mention of antepartum condition |
| ICD9dx64622 | Unspecified renal disease in pregnancy, without mention of hypertension, delivered, with mention of postpartum complication |
| ICD9dx64624 | Unspecified renal disease in pregnancy, without mention of hypertension, postpartum condition or complication |
| **Epilepsy** | |
| ICD9dx345 | Epilepsy and recurrent seizures |
| ICD9dx3451 | Generalized convulsive epilepsy |
| ICD9dx3454 | Localization-related (focal) (partial) epilepsy and epileptic syndromes with complex partial seizures |
| ICD9dx3459 | Epilepsy unspecified |
| ICD9dx34500 | Generalized nonconvulsive epilepsy, without mention of intractable epilepsy |
| ICD9dx34501 | Generalized nonconvulsive epilepsy, with intractable epilepsy |
| ICD9dx34510 | Generalized convulsive epilepsy, without mention of intractable epilepsy |
| ICD9dx34540 | Localization-related (focal) (partial) epilepsy and epileptic syndromes with complex partial seizures, without mention of intractable epilepsy |
| ICD9dx34541 | Localization-related (focal) (partial) epilepsy and epileptic syndromes with complex partial seizures, with intractable epilepsy |
| ICD9dx34550 | Localization-related (focal) (partial) epilepsy and epileptic syndromes with simple partial seizures, without mention of intractable epilepsy |
| ICD9dx34551 | Localization-related (focal) (partial) epilepsy and epileptic syndromes with simple partial seizures, with intractable epilepsy |
| ICD9dx34560 | Infantile spasms, without mention of intractable epilepsy |
| ICD9dx34561 | Infantile spasms, with intractable epilepsy |
| ICD9dx34570 | Epilepsia partialis continua, without mention of intractable epilepsy |
| ICD9dx34571 | Epilepsia partialis continua, with intractable epilepsy |
| ICD9dx34580 | Other forms of epilepsy and recurrent seizures, without mention of intractable epilepsy |
| ICD9dx34581 | Other forms of epilepsy and recurrent seizures, with intractable epilepsy |
| ICD9dx34590 | Epilepsy, unspecified, without mention of intractable epilepsy |
| ICD9dx34591 | Epilepsy, unspecified, with intractable epilepsy |
| **Other current conditions complicating pregnancy** | |
| ICD9dx64891 | Other current conditions classifiable elsewhere of mother, delivered, with or without mention of antepartum condition |
