# Supplementary material for: Caesarean section trends in Catalonia between 2013 and 2017 based on the Robson classification system: A cross-sectional study
Source: PLoS One. 2020 Jun 16;15(6):e0234727. doi: 10.1371/journal.pone.0234727 (PMC7297373; doi:10.1371/journal.pone.0234727)
Supplement: S2 Table — (DOCX) [file pone.0234727.s003.docx]

**Table S2** Bivariate analysis of socio-demographic and obstetric characteristics and type of delivery
