# Supplementary material for: Caesarean section trends in Catalonia between 2013 and 2017 based on the Robson classification system: A cross-sectional study
Source: PLoS One. 2020 Jun 16;15(6):e0234727. doi: 10.1371/journal.pone.0234727 (PMC7297373; doi:10.1371/journal.pone.0234727)
Supplement: S3 Table — (DOCX) [file pone.0234727.s004.docx]

**Table S3** Time trends in caesarean section rates including the variable BMI by groups of Robson

† Adjusted for maternal age, nationality, BMI, socioeconomic status, healthcare facility, pregnancy complication, delivery year and modified Robson classification.

• Adjusted for maternal age, nationality, BMI, socioeconomic status, healthcare facility, pregnancy complication and delivery year.

*p-value <0.05

**p-value <0.01

***p-value<0.001
